# A comparison between internal protein nanoenvironments of α-helices and β-sheets

**DOI:** 10.1371/journal.pone.0244315

**Published:** 2020-12-30

**Authors:** Ivan Mazoni, Jose Augusto Salim, Fabio Rogerio de Moraes, Luiz Borro, Goran Neshich

**Affiliations:** 1 Computational Biology Research Group, Embrapa Agricultural Informatics, Campinas, SP, Brazil; 2 Research Center on Biodiversity and Computing (BIOCOMP), Polytechnic School of the University of São Paulo (USP), São Paulo, Brazil; 3 Physics Department, Institute of Biosciences, Languages, and Exact Sciences (IBILCE), São Paulo State University (Unesp), Ribeirão Preto, SP, Brazil; 4 IoT & AI Solutions, CPqD Foundation, Campinas, SP, Brazil; Nanyang Technological University, SINGAPORE

## Abstract

Secondary structure elements are generally found in almost all protein structures revealed so far. In general, there are more β-sheets than α helices found inside the protein structures. For example, considering the PDB, DSSP and Stride definitions for secondary structure elements and by using the consensus among those, we found 60,727 helices in 4,376 chains identified in all-α structures and 129,440 helices in 7,898 chains identified in all-α and α + β structures. For β-sheets, we identified 837,345 strands in 184,925 β-sheets located within 50,803 chains of all-β structures and 1,541,961 strands in 355,431 β-sheets located within 86,939 chains in all-β and α + β structures (data extracted on February 1, 2019). In this paper we would first like to address a full characterization of the nanoenvironment found at beta sheet locations and then compare those characteristics with the ones we already published for alpha helical secondary structure elements. For such characterization, we use here, as in our previous work about alpha helical nanoenvironments, set of STING protein structure descriptors. As in the previous work, we assume that we will be able to prove that there is a set of protein structure parameters/attributes/descriptors, which could fully describe the nanoenvironment around beta sheets and that appropriate statistically analysis will point out to significant changes in values for those parameters when compared for loci considered inside and outside defined secondary structure element. Clearly, while the univariate analysis is straightforward and intuitively understood, it is severely limited in coverage: it could be successfully applied at best in up to 25% of studied cases. The indication of the main descriptors for the specific secondary structure element (SSE) by means of the multivariate MANOVA test is the strong statistical tool for complete discrimination among the SSEs, and it revealed itself as the one with the highest coverage. The complete description of the nanoenvironment, by analogy, might be understood in terms of describing a key lock system, where all lock mini cylinders need to combine their elevation (controlled by a matching key) to open the lock. The main idea is as follows: a set of descriptors (cylinders in the key-lock example) must precisely combine their values (elevation) to form and maintain a specific secondary structure element nanoenvironment (a required condition for a key being able to open a lock).

## Introduction

In a previous study of α-helices’ nanoenvironments, we presented data which clearly identify the most relevant protein structure attributes/descriptors/parameters fully describing the corresponding nanoenvironment [1]. Even though the univariate analysis has a lower success rate in determining (fully describing in a unique way) the nanoenvironment in question, this approach is however capable of pointing at those descriptors of the STING_RDB which are more relevant to fully describe it. In the case of proteins designated as all-α, the following descriptors had more than 80% of cases for p-value being lower than 1e-6: “hydrogen bond between main chain main chain atoms” (85.71%), “hydrogen bond between main chain main chain atoms-weighted neighbor averages by distance” (85.71%) and “hydrogen bond between main chain main chain” atoms weighted neighbor averages at surface” (82.85%). In the α+β case, only one descriptor had more than 80% of the p-value < 1e-6: “hydrogen bond between main chain—main chain atoms weighted neighbor averages by distance” (84.09%).

Considering that the total number of β-sheets found in the current PDB is far greater than the total number of α-helices in all proteins, we are interested in investigating not only the differences between α-helical and β-sheet nanoenvironments but also whether the precision and coverage of defining respective nanoenvironments are greater in the latter case.

It is also important to distinguish the two flavors of the β-sheets; they could be parallel or anti-parallel. In addition, β-sheets could even appear (rarely) in a form where only one strand exists, as one could verify in protein structures described in PDB entry: 1A2J. Within PDB files, one could easily locate the information about the strands at the secondary structure section. Parallel strands are identified by code number 1, and the anti-parallel strands are identified by code number -1. Single-stranded β-sheets are identified by code number 0 [2].

## Materials and methods

The structural and physical chemical parameters for analysis reported in this work from the STING_RDB [3].

The STING_RDB had 11,964,252,982 records in 98 tables (data from February 1, 2019). All of the raw data, ready for type of processing that we did in this paper, are available in two CSV files at: https://doi.org/10.6084/m9.figshare.13366814.v1 and https://doi.org/10.6084/m9.figshare.13369541.v1.

In the first step of the data preparation, we identified 121,757 structures annotated as all-β or α+/β in STING_RDB and for the selected structures we extracted their physical, chemical and structural descriptors. The number of protein structures identified was 2,862 for all-β and 118,895 for β-sheet in (α + β) + (α / β). To compare the internal nanoenvironment between α-helices and β-sheets, we selected the subset of 67 descriptors from all protein descriptors available in STING_RDB, resulting in descriptors from ten different classes (Table 1). In the Contacts class, “hb” acronym means hydrogen bond; “m” means main chain; “s” means side chain; “w” means water; “ch” means charge.

**Table 1:** STING_RDB descriptors used for the statistical analysis of the β sheet nanoenvironment.

Descriptor Class	Descriptor Name	#
Surface Accessibility	Accessible_Protein Surface_in_Isolation	1
Contacts	hb-mm	2
	hb-mwm	3
	hb-mwwm	4
	hb-ms	5
	hb-mws	6
	hb-mwws	7
	hb-ss	8
	hb-sws	9
	hb-swws	10
	hydrophobic	11
	aromatic	12
	ch_attractive	13
	ch_repulsive	14
	disulfide	15
ContactsWNA	hb-mm_WNADist	16
	hb-mwm_WNADist	17
	hb-mwwm_WNADist	18
	hb-ms_WNADist	19
	hb-mws_WNADist	20
	hb-mwws_WNADist	21
	hb-ss_WNADist	22
	hb-sws_WNADist	23
	hb-swws_WNADist	24
	hydrophobic_WNADist	25
	aromatic_WNADist	26
	ch_attractive_WNADist	27
	ch_repulsive_WNADist	28
	disulfide_WNADist	29
	hb-mm_WNASurf	30
	hb-mwm_WNASurf	31
	hb-mwwm_WNASurf	32
	hb-ms_WNASurf	33
	hb-mws_WNASurf	34
	hb-mwws_WNASurf	35
	hb-ss_WNASurf	36
	hb-sws_WNASurf	37
	hb-swws_WNASurf	38
	hydrophobic_WNASurf	39
	aromatic_WNASurf	40
	ch_attractive_WNASurf	41
	ch_repulsive_WNASurf	42
	disulfide_WNASurf	43
Density	IFR_CA_3	44
	Internal_CA_3	45
Physical_Chemical_Param	Electrostatic_Potential_at_CA	46
	Electrostatic_Potential_Average	47
	Electrostatic_Potential_at_LHA	48
	Hydrophobicity_KDI	49
PhysicoChemicalAndGeometricWNA	Electrostatic_Potential_at_CA_WNADist	50
	Electrostatic_Potential_Average_WNADist	51
	Electrostatic_Potential_at_LHA_WNADist	52
	Electrostatic_Potential_at_CA_WNASurf	53
	Electrostatic_Potential_Average_WNASurf	54
	Electrostatic_Potential_at_LHA_WNASurf	55
Space_Clash	Clash	56
	Percent	57
Structural_param	Cross_Link_Order_CA	58
	Cross_Pres_Order_CA	59
	Dihedral_Chi1	60
	Dihedral_Chi2	61
	Dihedral_Chi3	62
	Dihedral_Chi4	63
	Temperature_Factor_CA	64
UnusedContact	Number_Unused_Contact	65
UnusedContactWNA	Number_Unused_Contact_WNADist	66
	Number_Unused_Contact_WNASurf	67

Descriptors #2–10 refer to hydrogen bonds (hb) between main chain atoms (lines 2, 3 and 4), main chain and side chain atoms (lines 5, 6 and 7) and side chains atoms (lines 8, 9 and 10) of two amino acid residues, with no water molecule intervention, one water or two water molecules included (w or ww). Descriptors #16–43 refer to the same contact descriptors as above; however they are weighted by neighboring distances (lines 16–29) and weighted by surface distances (lines 30–43). Descriptors #44–45 refer to molecular density at the protein interface (line 44) and internal protein structure density (line 45). Descriptors #46–48 refer to electrostatic potential at the α-carbon (line 46), average value over residue atoms (line 47) and ep value at the last heavy atom (line 48). Descriptor #49 refers to hydrophobicity using the Kyte-Doolittle scale. Descriptors #50–55 refer to electrostatic potential descriptors, weighted by the neighboring residue distance and surface. Descriptors #56–57 refer to the number of clashes among residues and percent of clashes, respectively. Descriptors #58–64 refer to structural parameters such as cross link (line 58) and cross presence (line 59) order. The dihedral angles CHI (lines 60–63) and temperature factor at α-carbon (line 64). Descriptor #65 refers to the number of unused contacts, i.e., the difference between the maximum number of contacts available and the number of contacts established. Finally, descriptors #66–67 refer to the number of unused contacts, weighted by the neighboring residue distance (line 66) and surface (line 67). Classes terminated by WNA means Weighted Neighbor Averages; that can be by Distance (WNADist) or at corresponding Surface (WNASurf).

In the second step of data preparation we eliminated primary structure redundancy using the software CD-HIT [4]. In such a way, we first made the datamart that has all existing structures for which the primary sequences do not have a similarity greater than 95%. The second datamart has the structures for which the primary sequence do not have a similarity greater than 75% and, the third datamart, only structures that do not have sequence similarities greater than 50%, remained.

The third step in the data preparation process grouped the structures based on the PDB, DSSP and Stride secondary structure definition for β-sheet consensus. The consensus with maximum restriction is the one where the β-sheet initiates at the same residue number and has the same number of residues in all three definitions. Other possible consensuses are those between the PDB-DSSP, PDB-Stride and DSSP-Stride.

Although the second and third steps decreased the number of eligible structures to be used for analysis in this work, they produced a trusty non-redundant dataset.

The fourth step consisted of aligning the β sheets of identical lengths. In this work, the analysis of nanoenvironment for selected protein secondary structure element (PSSE) will be including, as a comparative non PSSE, the region of 32 residues before the N-terminus of the beta sheet element and 32 residues after the β-sheet C-terminus, just as was done in our study of alpha helical secondary structure elements. Aligned residues (part of the whole secondary structure elements) were used to evaluate the constructed nanoenvironment.

The fifth step consisted of calculating the average value and standard deviation for each selected parameter/descriptor at each position of the aligned secondary element structures. In that calculation, we obtained two sets of values: those corresponding to inside and outside of the β-sheet extension. We used these two separate sets to analyze our hypothesis. The hypothesis is that the descriptor values inside the β-sheet region are significantly different from the descriptor values outside the β-sheet region, considering in fact the 32 residues before and 32 residues after it as an “outside” domain.

We applied the Kolmogorov-Smirnov [5] test and MANOVA multivariate analysis [6] to verify the hypothesis. The data were prepared by selecting the structures containing β-sheets and grouping calculated average values for parameters selected in two sets: the “inside” domain of a secondary structure element and the one designated as an “outside” domain. The tests were applied using an R script.

The Kolmogorov-Smirnov test is a univariate test. For each descriptor in Table 1, we searched for group of the beta sheet elements having a same strand length and for all those which are available under such restriction, applied the test.

In contrast to the Kolmogorov-Smirnov test, MANOVA is a multivariate test. Consequently, we selected all descriptors in Table 1 for each group of distinctive available strand length and then applied the test for this sub-dataset.

The MANOVA test assumes that the data have a normal distribution. Hence, the preliminary step was undertaken to prepare the data satisfying such conditions. Therefore, we submitted the entry data to the Shapiro test [7], used to indicate which data have a normal distribution. Additionally, another step was introduced in order to achieve more precise MANOVA analysis: elimination of all descriptors correlated with each other. Both tests were performed using R scripts.

## Results

Fist we should describe here the volume of data prepared for statistical analysis. Considering the most restrictive consensus for definition of secondary structure element, i.e. the one with coinciding/equivalent PDB, DSSP and Stride definitions, and no redundancy (sequence wise similarity eliminated at indicated levels), there are 106,651 β-sheet elements in the all-β dataset and 167,080 β-sheet elements in (α + β) + (α / β) dataset. Table 2 shows the length of number of β sheets in terms of amino acid residues and the number of corresponding β sheet structures in all β and (α + β) + (α / β) datasets.

**Table 2:** Number of β-sheet elements (ordered by their length) for all-β and β in (α + β) + (α / β) structures.

	# of β-sheet elements	
# AAR	all-β	β-sheet in (α + β) + (α / β)
5	22663	35354
6	21926	34490
7	16996	27055
8	14112	21907
9	10502	16110
10	8295	12765
11	4849	7947
12	2944	4337
13	1601	2606
14	955	1529
15	843	1258
16	293	509
17	214	378
18	111	248
19	153	269
20	65	89
21	45	75
22	38	48
23	20	39
24	12	30
25	4	9
26	4	11
27	3	6
28	0	1
30	0	1
31	0	1
33	3	7
36	0	1

### Univariate tests for the all-β dataset

**Fig 1:**
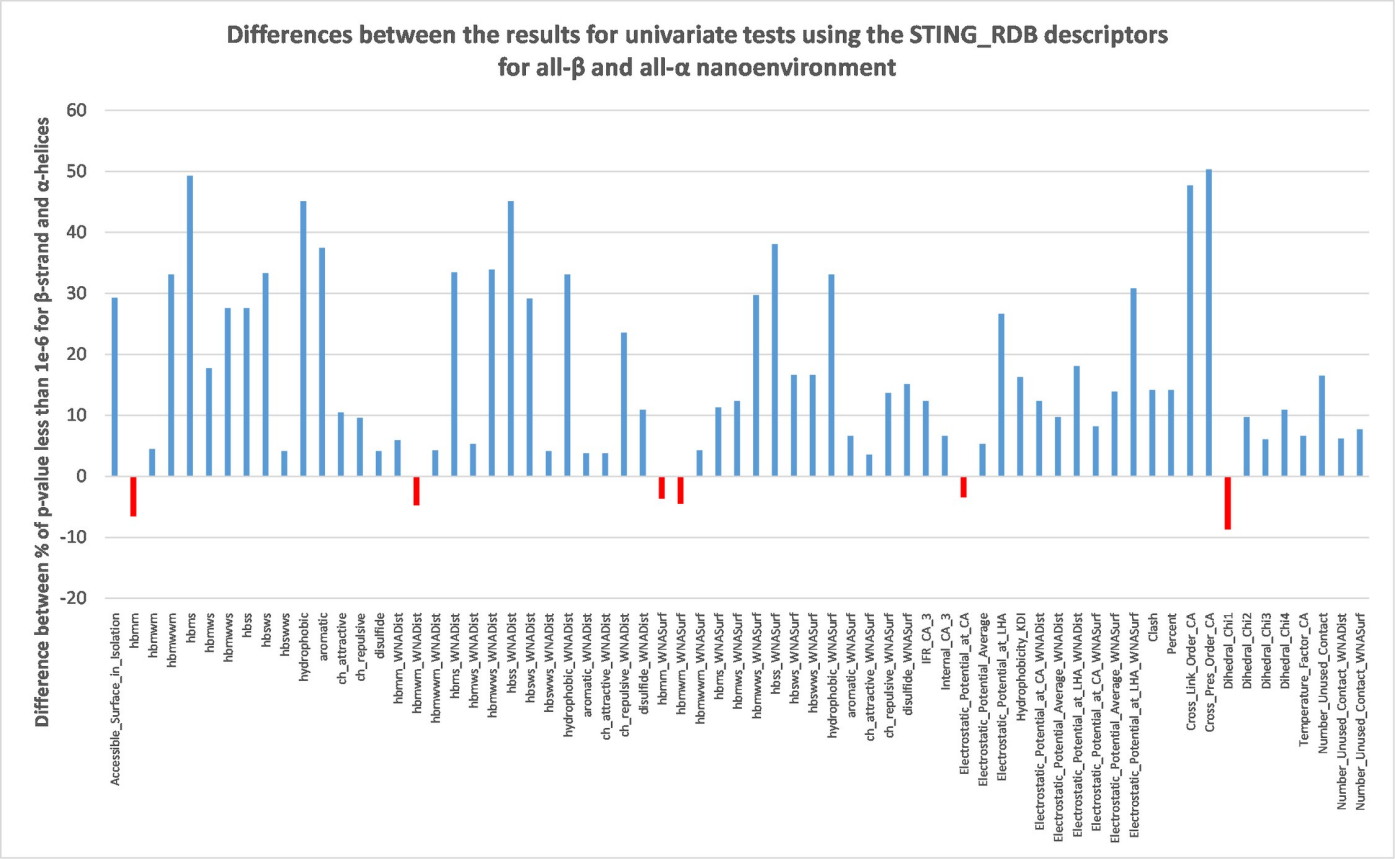
Differences observed in percentwise coverage for p-values being less than 1e-6 for univariate test results using the STING_RDB descriptors for beta sheets in all-β and alpha helices in all-α protein nanoenvironments.

The univariate test is used here merely as the application of classical hypotheses testing, where a single structure descriptor is used as the main driver of effect (in this case: a formation or existence of particular secondary structure element). From Table 2, we have 24 distinct β-sheet lengths identified within the all-β dataset. Considering the 67 preselected STING_RDB descriptors (Table 1), 1,608 tests were applied in total (one for each of 67 descriptors and one for each of 24 distinct β-sheet secondary structure element lengths). For 881 tests, the p-value calculated was less than 1e-6. This means that for 54.79% of cases, we are confident in accepting the initial hypothesis that the descriptors “inside” the β-sheet element are significantly different from the corresponding values for the same descriptors for the region “outside” the β-sheet element. The following descriptors demonstrated p-values being lower than 1e-6 for more than 80% of cases: “hbmm_WNADist” (91.66%), “Cross_Pres_Order_CA” (87.50%) and “Number_Unused_Contacts_WNADist” (83.33%). See Fig 1 for the distribution of values calculated for all descriptors.

To gain more precision, we applied the same Kolmogorov-Smirnov test but now for two separate datamarts: the all-β proteins with subtype: parallel only, and for all-β proteins, subtype: anti-parallel only. Additionally, we introduced the third subtype: all-β proteins with only one β-sheet strand.

For the all-β proteins, subtype: parallel only dataset, we have 2,345,886 strands. For this subtype, we identified 23 distinct β-sheet lengths (from Table 2, no strands for length 27). Considering the same 67 STING_RDB descriptors used, 1,541 tests were applied in total. We obtained 38.74% of cases with p-values less than 1e-6. No descriptor had more than 80% coverage having p-value being < 1e-6. The four descriptors considered as the best cases in this particular analysis were just above 60% coverage level: “hbmm_WNADist” (65.21%), “Cross_Pres_Order_CA” (65.21%), “Hydrophobicity_KDI” (60.86%) and “Cross_Link_Order_CA” (60.86%). See S1 Fig in the Supplementary Material for values corresponding to all selected descriptors.

At the same time, for all-β proteins, subtype: anti-parallel dataset, we identified 3,493,141 strands. Considering the 24 β-sheet lengths identified within the all-β dataset and the 67 descriptors, we have 1,608 tests applied in total. We obtained a total of 54.16% cases with a p-value less than 1e-6. The descriptors with p-values being < 1e-6 are some of the same (two out of three) as those found in corresponding tests for all-β proteins, subtype: parallel only: “hbmm_WNADist” (91.66%), ‘Cross_Pres_Order_CA” (87.50%) and “Number_Unused_Contacts_WNADist” (83.33%). See Fig 1 for all descriptor distributions.

A β sheet with only one strand is a particular case of a β sheet structure element. We have 51 structures with 1 strand only in the all-β dataset, with 12 distinct lengths. These lengths multiplied by the 67 STING_RDB descriptors (from Table 1) result in 804 tests. For the tests done, 8.83% cases had a p-value less than 1e-6. The 38 descriptors, representing 59.71% of the STING_RDB descriptors used in the Kolmogorov-Smirnov test, do not have a p-value < 1e-6. The best cases we could cite here were for “Accessible_Surface_in_Isolation” and “hbmm_WNADist”, both with 50.00% coverage.

Table 3 demonstrates above described results.

**Table 3:** Results of the Kolmogorov-Smirnov test applied to the all-β dataset.

Dataset	Number of tests	p-value < 1e-6 [%]
all-β: all subtypes	1,608	54.79
all-β parallel only	1,541	38.74
all-β anti-parallel only	1,608	54.16
all-β 1 strand only	804	8.83

In Fig 1, we can see the percentwise differences between the values for coverage of p-value being less than 1e-6 for descriptors used to describe nanoenvironments of beta sheet in all-β type of proteins and alpha helix in the all-α type of proteins.

### Univariate tests for β in the (α + β)+ (α/β) dataset

According to Table 2, we found28 distinct β-sheet lengths in (α + β) + (α / β) type of protein structures. That results in 1,876 tests. The portion of p-values being < 1e-6 was in this case 49.57%, which is less than in the previously analyzed all-β case (54.79%). This is mostly expected observation as we have in this case two PSSE types instead of only one.

**Fig 2:**
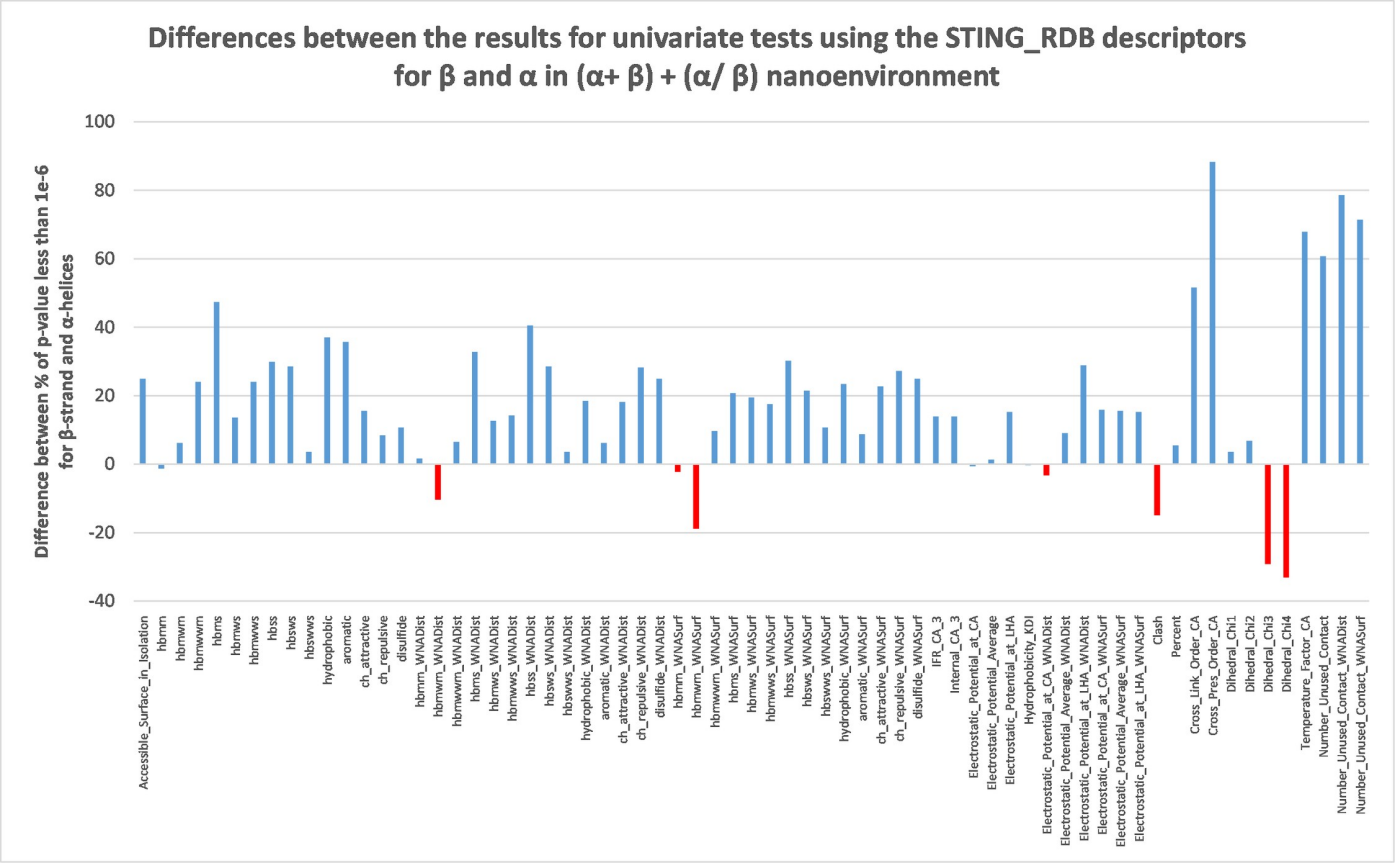
Differences observed in percentwise coverage for p-values being less than 1e-6 for univariate test results using the STING_RDB descriptors for β-sheets in (α + β)+(α / β) protein types and for α-helices in (α + β) + (α / β) proteins nanoenvironment.

For the dataset of β-sheets in (α + β) + (α / β) type of proteins, the following descriptors had more than 80% of p-values being < 1e-6: “Cross_Pres_Order_CA’ (92.85%), “hbmm_WNADist” (85.71%) and “Cross_Link_Order_CA” (85.71%). Fig 2 shows the difference in p-value coverage for descriptors examined by Kolmogorov-Smirnov tests for β-sheet in α + β protein type and α-helix in α + β protein type structures.

For β-sheet in (α + β) + (α / β) type of proteins, the parallel only subtype has 23 distinct strand lengths (no strands for lengths 27, 28, 30, 31 and 36 amino acids), resulting in 1,541 tests., which 41.01% of the tests had a p-value < 1e-6. The best result was for “Cross_Pres_Order_CA” (73.91%). The other descriptors showed a coverage being under 70% for p-value lower than 1e-6.

For β-sheet in (α + β) + (α / β) type of proteins, the anti-parallel only subtype has strands with the lengths described in Table 2. Considering the 67 STING_RDB descriptors from Table 1, we have run 1,876 tests. In the tests done, the portion of p-value being < 1e-6 was 49.41%. Three descriptors showed coverage above 80%: “Cross_Pres_Order_CA” (92.85%), “Cross_Link_Order_CA” (85.71%) and “hbmm_WNADist” (82.14%).

For β-sheet in (α + β) + (α / β) type of proteins, with one strand only, we have 17 distinct lengths: 5, 6, 7, 8, 9, 10, 11, 12, 13, 14, 15, 16, 17, 19, 21, 23 and 25, resulting in 1,139 possible tests. The portion of p-value being < 1e-6 was 14.04%. In this case, the best result was for the descriptor: “hbmm_WNADist (70.58%)”. All the other descriptors showed p-value lower than 1e-6 coverage in under 60% cases.

Table 4 presents the results of the Kolmogorov-Smirnov test for β in α + β.

**Table 4:** Results of the Kolmogorov-Smirnov test applied to β-sheets in the (α + β) + (α / β) protein dataset.

Dataset	Number of tests	p-value < 1e-6 [%]
β-sheet in (α + β) + (α / β) all subtypes	1,876	49.57
β-sheet in (α + β) + (α / β) subtype parallel only	1,541	41.01
β-sheet in (α + β) + (α / β) subtype anti-parallel only	1,876	49.41
β-sheet in (α + β) + (α / β) subtype one strand only	1139	14.04

In the Supplementary Material, we present the plots for descriptors used with a p-value < 1e-6 in all univariate tests.

## Manova

Previous work indicated that the Kolmogorov-Smirnov test is partially useful but certainly not the best way to analyze the nanoenvironment of a secondary structure element [1]. As demonstrated in Table 3, at best, we found 54.79% of analyzed cases with a p-value < 1e-6. That obviously happens because univariate tests consider only one descriptor, while the studied nanoenvironments are built by all interacting forces, fully described only with a complete set of descriptors. Thus, we applied the MANOVA test to the same group of above described datasets. Table 5 shows the results for the four MANOVA statistical tests available in the R programming language: Pillai, Wilks, Hotelling-Lawley and Roy [8].

**Table 5:** Results of the MANOVA test applied to the all-β protein type dataset.

Dataset	Number of tests	Test	p-value < 1e-6 [%]
all-β: all subtypes	13	Pillai	76.92
		Wilks	76.92
		Hotelling-Lawley	76.92
		Roy	76.92
all-β: subtype parallel only	9	Pillai	77.77
		Wilks	77.77
		Hotelling-Lawley	77.77
		Roy	77.77
all-β: subtype anti-parallel only	14	Pillai	78.57
		Wilks	78.57
		Hotelling-Lawley	85.71
		Roy	78.57
all-β: subtype 1 strand only	3	Pillai	100.00
		Wilks	100.00
		Hotelling-Lawley	100.00
		Roy	100.00

For all-β type proteins, we have 24 β-sheet distinct lengths (Table 2). After removing the data with no normal distribution, and eliminating correlated descriptors, 13 tests were applied. Next, we present each STING_RDB descriptor and between parentheses a number indicating in how many tests each of them was used: “hbmwwm_WNADist” (6), “hydrophobic_WNASurf” (4), “hydrophobic_WNADist” (4), ‘hbmwws_WNADist” (4), ‘hbmwm_WNADist’ (3), “ch_attractive” (3), “Number_Unused_Contact” (2), “hbswws_WNADist” (2), “hbmwws_WNASurf” (2), “hbmwws” (2), “hbmws_WNASurf” (2), “hbmws_WNADist” (2), “Electrostatic_Potential_at_LHA_WNASurf” (2), “Electrostatic_Potential_at_LHA” (2), “Dihedral_Chi(4)” (2), “Dihedral_Chi(3)” (2), “ch_repulsive” (2), “Number_Unused_Contact_WNASurf” (1), “hbsws_WNADist” (1), “hbss_WNASurf” (1), “hbss” (1), “hbmwwm_WNASurf” (1), “hbmwwm” (1), “hbmwm_WNASurf” (1), “hbmm” (1), “Electrostatic_Potential_Average_WNASurf” (1), “Electrostatic_Potential_Average_WNADist” (1), “disulfide_WNASurf” (1), “Dihedral_Chi(2)” (1), “Dihedral_Chi(1)” (1), “Cross_Link_Order_CA” (1), “aromatic_WNASurf” (1), “aromatic_WNADist” (1). The Pillai, Wilks, Hotelling-Lawley and Roy tests returned results indicating coverage of 76.92% for p-values being < 1e-6. In comparison, the univariate tests applied to the same dataset had 54.79% coverage of p-value being < 1e-6.

For the all-β proteins, subtype: parallel only dataset, we identified 23 β-sheet lengths (from Table 2, no strands for length 27). After removing the non-normally distributed data and the correlated descriptors, 9 tests were applied. Next we present each STING_RDB descriptor and between parentheses a number indicating in how many tests each them was used: “hbmwwm_WNADist” (5), “hydrophobic_WNADist” (3), “hbmwws_WNADist” (3), “hbmws_WNASurf” (3), “disulfide_WNADist” (3), “Number_Unused_Contact_WNASurf” (2), “hydrophobic_WNASurf” (2), “hbswws_WNADist” (2), “hbmwwm_WNASurf” (2), “hbmwwm (2), “Electrostatic_Potential_at_LHA_WNASurf” (2), “disulfide_WNASurf” (2), “ch_attractive_WNADist” (2), “Temperature_Factor_CA” (1), “Number_Unused_Contact” (1), “IFR_CA” (1), “hbss_WNADist” (1), “hbmwws_WNASurf” (1), “hbmws_WNADist” (1), “hbms_WNASurf” (1), “Electrostatic_Potential_Average_WNASurf” (1), “Electrostatic_Potential_Average_WNADist” (1), “Electrostatic_Potential_at_LHA_WNADist” (1), “Electrostatic_Potential_at_LHA” (1), “Dihedral_Chi(4)” (1), “Dihedral_Chi(3)” (1), “Dihedral_Chi(1)” (1). The Pillai, Wilks, Hotelling-Lawley and Roy tests returned results indicating coverage of 77.77% for p-values being < 1e-6. The univariate tests applied to the same dataset had a p-value being < 1e-6 in 38.74% of cases.

For the all-β proteins, subtype: anti-parallel only dataset, we have 24 β-sheet lengths (Table 2). After removing the non-normally distributed data and the correlated descriptors, 14 tests were applied. Next we present each STING_RDB descriptor and between parentheses a number indicating in how many tests each of them was used: “hydrophobic_WNADist” (5), “hbmwwm_WNADist” (5), “hbmwws_WNADist” (4), “Dihedral_Chi(3)” (4), “hydrophobic_WNASurf” (3), “hbmwws_WNASurf” (3), “hbmwwm_WNASurf” (3), “hbmwm_WNADist” (3), “ch_repulsive_WNADist” (3), “Number_Unused_Contact” (2), “hbswws_WNADist” (2), “hbss (2), hbmwwm” (2), “hbmws_WNADist” (2), “hbmm” (2), “Electrostatic_Potential_at_LHA_WNADist” (2), “Electrostatic_Potential_at_LHA” (2), “ch_repulsive_WNASurf” (2), “ch_repulsive” (2), “ch_attractive” (2), “aromatic_WNASurf” (2), “Number_Unused_Contact_WNASurf” (1), “hbswws” (1), “hbsws_WNADist” (1), “hbsws” (1), “hbss_WNASurf” (1), “hbmwws” (1), “hbmws_WNASurf” (1), “hbmwm_WNASurf” (1), “hbms_WNASurf” (1), “hbmm_WNADist” (1), “Electrostatic_Potential_Average_WNASurf” (1), “Electrostatic_Potential_Average_WNADist” (1), “Electrostatic_Potential_at_LHA_WNASurf” (1), “disulfide_WNASurf” (1), “Dihedral_Chi(4)” (1), “Dihedral_Chi(2)” (1), “Dihedral_Chi(1)” (1), “Cross_Pres_Order_CA” (1), “Cross_Link_Order_CA” (1), “aromatic_WNADist” (1). The Pillai, Wilks, Hotelling-Lawley and Roy tests returned results indicating coverage of 78.57% for p-values being < 1e-6, and the Hotelling-Lawley test presents 85.71% of cases where p-values is < 1e-6. The univariate tests applied to the same dataset had 53.84% of p-value < 1e-6.

For the all-β proteins, subtype: one strand only dataset, we have 12 lengths: 5, 6, 7, 8, 9, 10, 11, 12, 13, 14, 15 and 16. After removing the non-normally distributed data and the correlated descriptors, 3 tests were applied. Next, we present each STING_RDB descriptor and between parentheses, a number indicating in how many tests each one of them was used: “hbmm” (1); “hbmm_WNADist” (1); “hbmwm_WNASurf” (1); “hbmws_WNASurf” (1); “hbmwwm_WNASurf” (1); “Number_Unused_Contact_WNADist” (1). The Pillai, Wilks, Hotelling-Lawley and Roy tests returned results indicating 100.0% coverage for p-values being < 1e-6. The univariate tests applied to the same dataset had an 8.84% p-value < 1e-6.

We repeated the MANOVA test for β-sheets in the (α + β) + (α / β) protein dataset. Table 6 shows the results.

**Table 6:** Results of the MANOVA test applied to β in the (α + β) dataset.

Dataset	Number of tests	Test	p-value < 1e-6 [%]
β-sheet in (α + β)+(α / β): all subtypes	12	Pillai	83.33
		Wilks	83.33
		Hotelling-Lawley	83.33
		Roy	83.33
β-sheet in (α + β)+(α / β): subtype parallel only	6	Pillai	83.33
		Wilks	83.33
		Hotelling-Lawley	83.33
		Roy	83.33
β-sheet in (α + β)+(α / β): subtype anti-parallel only	11	Pillai	81.81
		Wilks	81.81
		Hotelling-Lawley	81.81
		Roy	81.81
β-sheet in (α + β)+(α / β): subtype 1 strand only	0	Pillai	-
		Wilks	-
		Hotelling-Lawley	-
		Roy	-

## Conclusions

The Kolmogorov-Smirnov hypothesis test demonstrated that interatomic contacts among amino acid residues are essential for maintaining the existence of β sheets and are therefore crucial in characterization of beta sheet nanoenvironment. In the case of antiparallel all-β proteins, the descriptor “hbmm_WNADist” (number of hydrogen bonds established among neighboring main chains, weighted by the distance from the neighboring residues) had a p-value lower than 1e-6 in 91.67% of the tests. Therefore, this particular descriptor qualifies here as the most relevant nanoenvironment descriptor, or MRND. For proteins of the type β-sheet in (α + β) + (α / β), the descriptor “Cross_Pres_Order_CA” (the number of contacts of so called Cross Presence Order type which might also be interpreted as an indicator for possible contacts among sequence wise not neighboring amino acid residues) had a p-value lower than 1e-6 in 92.85% of the cases.

However, as we analyzed in our previous work (MAZONI, 2018), univariate tests are not the most suitable for this type of analysis because the maintenance of a nanoenvironment happens through a fine tuning of a set of parameters, acting at the same time, just as previously explained in analogy with key-lock cylinders In the case of β sheets, the univariate tests achieved a rate of approximately 51% coverage (we define coverage here as a % of cases where the p-value was calculated as being <1e-6, and therefore, making possible acceptance of the hypothesis we started with). That means that in 51% of the studied cases, the values ​​of the descriptors for those residues present within the β sheet are significantly different from the values ​​of the descriptors of residues outside that specific neighborhood. This hit rate rises to almost 85% in the case of the MANOVA multivariate test. With such a simple fact it is clear that the set of the MRND must be sought among best performing descriptors in MANOVA tests.

MANOVA tests have shown that different types of interatomic contacts among amino acid residues are essential (MRNDs) for maintaining the existence of β sheets and characterizing the nanoenvironment here analyzed. In the case of all-β proteins, the following descriptors were selected in more than 30% of cases where an initial hypothesis was accepted: “hbmwwm_WNADist” (46.15%), “hbmwws_WNADist” (30.77%), “hydrophobic_WNADist” (30.77%), and “hydrophobic_WNASurf” (30.77%). For β-sheet in (α + β) + (α / β) proteins, the “hbmwws_WNADist” descriptor was used in more than 30% of cases (30.77%) where an initial hypothesis was accepted. The other descriptors, although they contributed to the success rate of MANOVA tests, approaching 85%, were selected less often.

When comparing the MRND of the beta sheets nanoenvironment with the one where α helices are present (MAZONI, 2018), we conclude that these are two quite different nanoenvironments. In the case of helices, the descriptors “Number_Unused_Contacts” (potential for interatomic contact formation), “Electrostatic_Potential” and number of contacts of “Hbms” type (hydrogen bonds between main and side chains) are crucial there. For the β sheets, however, we see some othertypes of descriptors being pointed out as the MRND, mostly contacts of weighted by the distance from neighboring residues type; “hbmwwm_WNADist”, “hbmwws_WNADist”, “hydrophobic_WNADist”, “hydrophobic_WNASurf”.

Clearly, the nature of analysis used here as well as the concept of MRND permits us here to rationalize the meaning of two MRND groups, respective to alpha helical and beta sheet nanoenvironment. While the first group undoubtable points out to more local interactions as crucial for alpha helical nanoenvironment, the latter definitely points toward more non local interactions, involving also sequence wise non neighboring residues.

In this work, we further expanded our general understanding of nanoenvironments, including into consideration both α helices (previously published) and now, β sheets.

Nanoenvironments exist to provide favorable conditions for the secondary structure formation and maintenance (in this case, the beta-sheets) within forming and/or already formed full protein structure. The importance of the nanoenvironment concept is very usable in a general understanding of the proteins structures, possible standardization of attributes describing structural and functional districts and consequently, enabling investigation of a quality of those structures. It is also important for understanding the relationship between primary sequence, tertiary structure and protein function. Most important aspect of all this would be consideration of observed structural promiscuity given the variation in terms of content that a primary sequence demonstrate at one end, then observed small variations in structure and resulting larger variations in function on the other end. The concept of nanoenvironment makes such observed behavior more intuitively understandable, once we grasp the importance of the fact that a composite descriptor environment generally is not changing much as the localized sequence variations occur. Yet some other sequence changes, critical to resulting composite descriptor environment, cause its modifications, which might be sufficiently large in order to promote functional change.

The natural next step in a series of research undertakings we are conducting, aiming to reveal the MRND of PSSE, would be to analyze in detail the nanoenvironment where are formed different types of turns.

At last but not the least, it is worth mentioning here that the current results contribute to our collection in the Dictionary of Internal Protein Nanoenvironments (DIPN) descriptors, the repository of all MRND for 10 most studied protein nanoenvironments. This repository is already available in its preliminary, not yet fully populated form at: https://www.proteinnanoenvironments.cnptia.embrapa.br/index.html

## Supporting information

S1 Fig Results for univariate tests using the STING_RDB descriptors for all-β parallel only, all-β anti-parallel only and one strand only nanoenvironment. (TIF)

S2 Fig Results for univariate tests using the STING_RDB descriptors for β-sheet in (α + β)+(α / β) parallel only, all-β anti-parallel only and one strand only nanoenvironment. (TIF)
