# Supplementary figures and images for: A comparison between internal protein nanoenvironments of α-helices and β-sheets
Source: PLoS One. 2020 Dec 30;15(12):e0244315. doi: 10.1371/journal.pone.0244315 (PMC7773245; doi:10.1371/journal.pone.0244315)

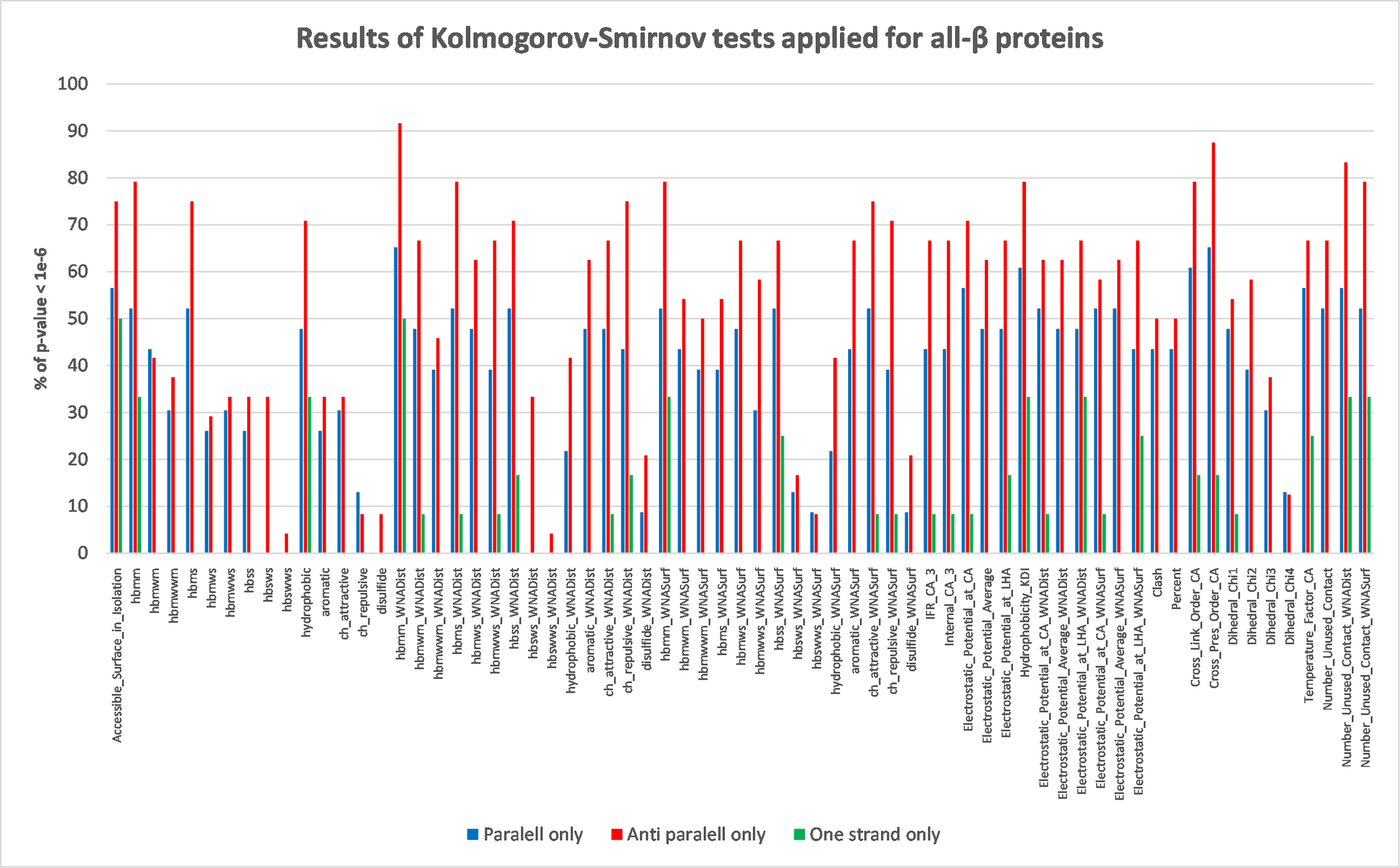

Supplement: S1 Fig — (TIF) [file pone.0244315.s001.tif]

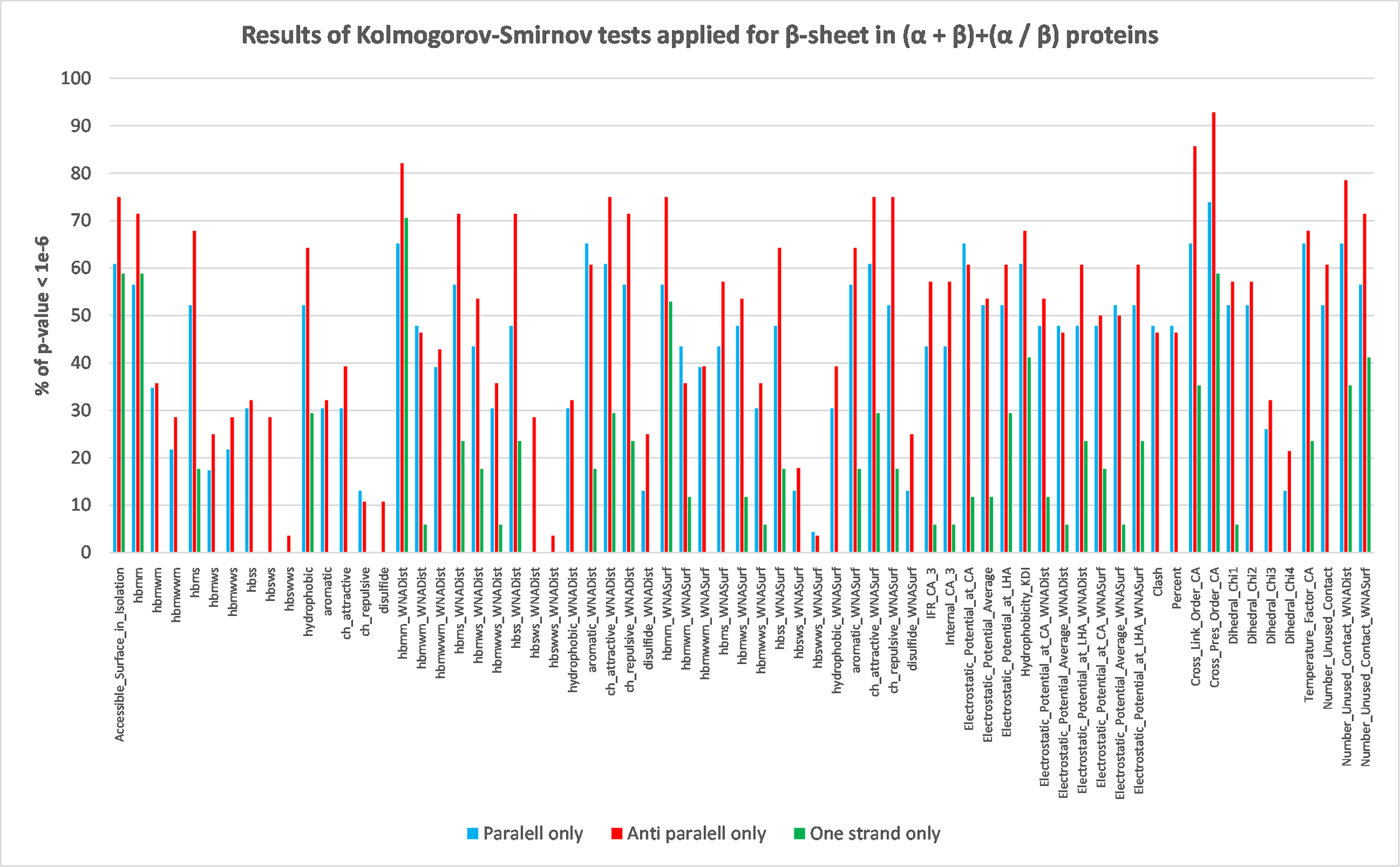

Supplement: S2 Fig — (TIF) [file pone.0244315.s002.tif]
